# Comparison of different degrees of varus deformity correction with open-wedge high tibial osteotomy: a retrospective study over 5 years

**DOI:** 10.1186/s13018-024-04557-7

**Published:** 2024-01-28

**Authors:** Kuishuai Xu, Tianrui Wang, Tengbo Yu, Xia Zhao, Yingze Zhang, Liang Zhang

**Affiliations:** 1 Department of Sports Medicine, The Affiliated Hospital of Qingdao University, Qingdao, 266000 Shandong China; 2 Department of Traumatology, The Affiliated Hospital of Qingdao University, Qingdao, 266000 Shandong China; 3 Institute of Sports Medicine and Health, Qingdao University, Qingdao, Shandong China; 4 Department of Orthopedic Surgery, Qingdao Hospital, University of Health and Rehabilitation Sciences (Qingdao Municipal Hospital), Qingdao, 266000 Shandong China; 5 Department of Abdominal Ultrasound, Affiliated Hospital of Qingdao University, Qingdao, 266000 Shandong China

**Keywords:** Open-wedge high tibial osteotomy, Overcorrection, Imaging, Functional recovery, Complications

## Abstract

**Objective:**

This study aims to investigate the clinical efficacy and complications associated with open-wedge high tibial osteotomy (OWHTO) in the treatment of medial compartment knee osteoarthritis. Additionally, the compensatory changes in the hip, patellofemoral, and ankle regions will be assessed through imaging.

**Methods:**

A retrospective analysis of clinical data pertaining to 86 patients who underwent OWHTO at the Affiliated Hospital of Qingdao University from January 2015 to September 2018 was conducted. The weight-bearing line ratio (WBLR) was measured postoperatively, and patients were categorized into a normal group (50% < WBLR ≤ 62.5%, *n* = 67) and an overcorrection group (WBLR > 62.5%, *n* = 19). Various parameters, including hip–knee–ankle angle (HKA), medial proximal tibial angle (MPTA), lateral distal femoral angle (LDFA), joint line convergence angle (JLCA), and posterior tibial slope (PTS), were measured before surgery and at the last follow-up to assess lower limb line correction. The compensatory changes in adjacent joints were evaluated by measuring hip abductor angle (HAA), tibial plafond inclination (TPI), talus inclination angle (TIA), Carton–Deschamps index, lateral patellar tilt (LPT), lateral patellar shift (LPS), medial patellofemoral space, and lateral patellofemoral space in both groups. The American Hospital for Special Surgery (HSS) score and the Western Ontario and McMaster Universities Osteoarthritis Index (WOMAC) of the affected knee were assessed before surgery and at the last follow-up, and the incidence of complications in both groups was analyzed.

**Results:**

Postoperative complications occurred in 26.32% (five cases) of the overcorrection group and 5.97% (four cases) of the normal group, with a statistically significant difference (*χ*2 = 4.548, *p* = 0.033). No significant differences were observed in HSS and WOMAC between the two groups at the last follow-up. HAA was − 2.44 ± 1.98° in the overcorrection group and − 1.16 ± 2.1° in the normal group, with a statistically significant difference (*t* = 2.32, *p* = 0.023). There were no significant differences in other imaging indexes.

**Conclusion:**

Overcorrection of varus deformity may not significantly impact clinical outcomes within 5 years post-OWHTO but may elevate the incidence of postoperative complications and lead to increased compensatory adduction of the hip.

## Introduction

OWHTO has emerged as a prevalent intervention for medial compartment osteoarthritis (OA) in recent years [1]. The pivotal determinant for sustained success post-OWHTO lies in redirecting the force line of the lower limb outward to achieve an equitable distribution of mechanical load within the knee joint [2]. The critical aspect of this procedure is the selection of the force line correction point. The literature suggests that optimal therapeutic outcomes are attainable through the application of the Fujisawa point, specifically the 62.5% point on the lateral tibial plateau, post-surgical correction [3]. Dugdale et al. [7] have proposed that the force line subsequent to osteotomy should traverse the region proximal to the lateral 60% of the tibial plateau on the coronal plane. Myrnerts [8], in pursuit of superior long-term results, has posited that patients subjected to excessive correction exhibit significantly better efficacy compared to those with normal correction. Consequently, there exists no unanimous consensus on the methodology for determining the target force line to optimize surgical outcomes.

The prevailing belief posits a correlation between the choice of alignment position and the condition of cartilage wear and meniscus. In instances of pronounced varus deformity of the knee joint, intraoperative correction of the osteotomy angle necessitates a larger angle. Consequently, varying corrective protocols is implemented for the medial compartment cartilage injury of the knee, contingent upon the diverse stages of knee osteoarthritis. Notably, it remains unexplored whether overcorrected knee joints following varus alignment lead to compensatory alterations in adjacent hip, patellofemoral, and ankle joints. Thus, we conducted a retrospective study spanning a 5-year duration to assess the clinical efficacy of OWHTO in addressing overcorrected varus deformity in the treatment of medial compartment knee osteoarthritis, utilizing imaging and clinical function scores. Additionally, an evaluation of compensatory changes in the hip, patellofemoral, and ankle joints was undertaken.

## Materials and methods

### Inclusion and exclusion criteria

A retrospective analysis encompassed 86 osteoarthritis patients who underwent unilateral open-wedge high tibial osteotomy (OWHTO) at the Affiliated Hospital of Qingdao University between January 2015 and September 2018. Inclusion criteria comprised a clinical diagnosis consistent with early anterior medial compartment osteoarthritis of the knee, a minimum follow-up period of 5 years, absence of neuromuscular disease, clear knee joint structure on X-ray examination, knee varus deformity < 15° (primarily proximal tibia deformity), flexion contracture < 10°, and knee motion > 110°. Exclusion criteria encompassed lateral intercompartment osteoarthritis of the knee joint or inflammatory arthritis, simultaneous OWHTO with other joint surgeries, bilateral OWHTO, incomplete or missing imaging data, knee ligament injury or insufficiency, and inadequate correction (weight-bearing line ratio [WBLR] ≤ 50%). Based on the inclusion/exclusion criteria, the 86 patients were categorized into two groups according to postoperative WBLR: the normal correction group (50% < WBLR ≤ 62.5%, *n* = 67) and the overcorrection group (WBLR > 62.5%, *n* = 19). All patients consented to participation, signing informed consent, and the study protocol gained approval from our hospital's Ethics Committee (QYFYWZLL26357).

### Operation technique

During the procedure, patients assumed a supine position under general anesthesia, with tourniquets routinely applied at the root of the thigh on the surgical side. A 5-cm incision was made to expose the medial patellar ligament, followed by successive cuts through the skin and subcutaneous tissue to reveal the superficial layer of the medial collateral ligament, which was then incised. Under fluoroscopy, two Kirschner wires were positioned 5 cm below the articular surface, directed toward the small head of the fibula. A high tibial osteotomy was executed along the trajectory of the Kirschner wires, with tibial bracing. The Tomofix locking plate was meticulously positioned and fixed under fluoroscopy to align with the pre-set point of the force line. The implanted iliac bone, exhibiting an angle greater than 15°, was braced, ensuring satisfactory internal fixation. The operative site was rinsed and sutured.

### Postoperative rehabilitation

Following surgery, the affected limb was elevated, and all patients underwent pressure bandaging with an elastic bandage. The drainage tube was clamped for 4 h postoperatively, subsequently opened. Standardized administration of antibiotics and anticoagulants occurred within the first 24 h after surgery. Isometric muscle contraction exercises commenced on the bed from the day after surgery. The drainage tube was removed based on the drainage flow, and flexion–extension exercises were initiated from a limited angle.

### Clinical assessment

All patients underwent preoperative and final knee function follow-up assessments, including: ① The American Hospital for Special Surgery score (100 points): pain (30 points), function (22 points), range of motion (18 points), muscle strength (10 points), flexion deformity (10 points), stability (10 points), and reduction items. A higher score reflects improved function; ② The Western Ontario and McMaster University Osteoarthritis Index (WOMAC): Utilizing a 0–4 scale, it evaluates five aspects of pain, two aspects of stiffness, and 17 aspects of difficulty in daily activities, with higher scores indicating poorer functioning. Postoperative complications were ascertained through outpatient review and telephone follow-up, encompassing neurovascular injury, lateral hinge fracture, infection, deep vein thrombosis of the lower extremity, delayed or non-union, and joint stiffness.

### Radiological evaluation

**Fig. 1:**
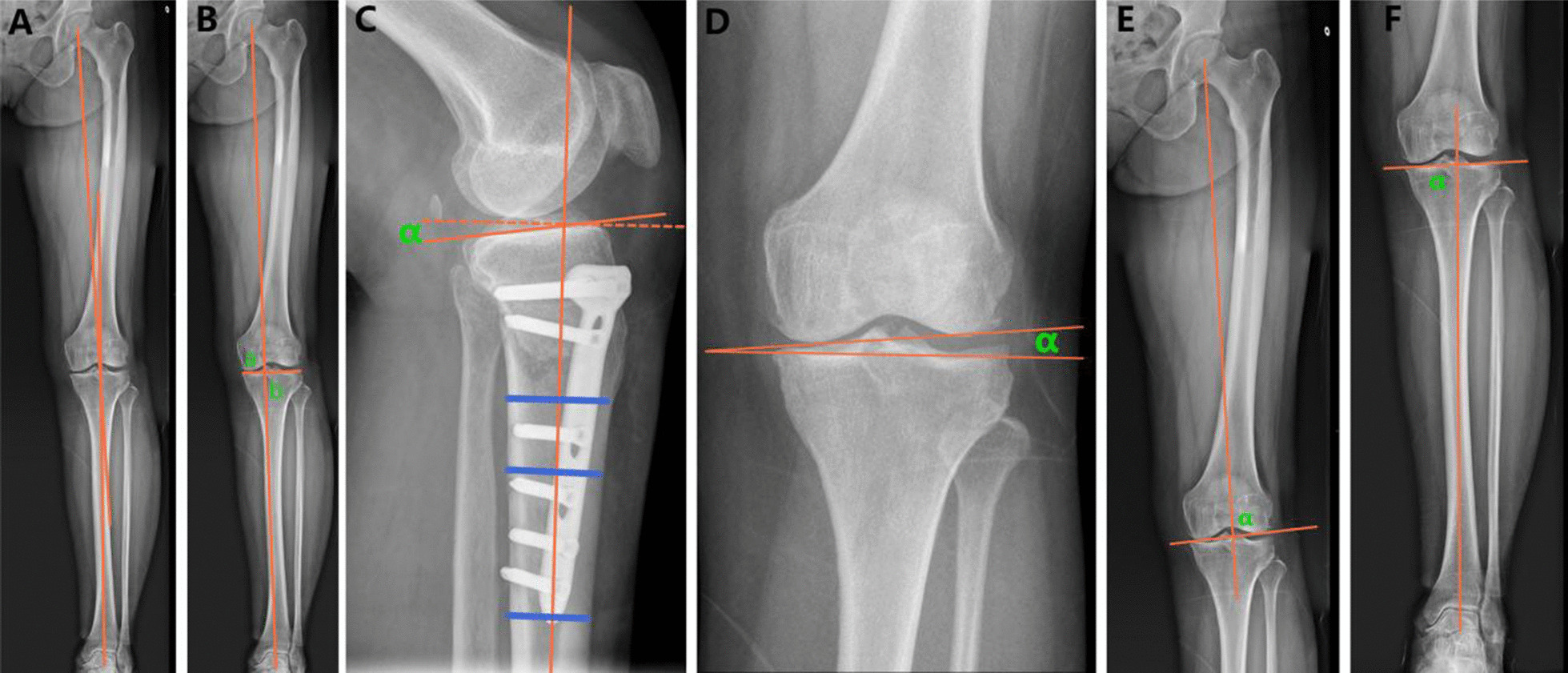
Measurement of imaging indexes related to lower limb force line. **A** Hip–knee–ankle angle (HKA). **B** Weight-bearing line ratio (WBLR). **C** Posterior tibial slope (PTS). **D** Joint line convergence angle (JLCA). **E** Distal lateral femoral angle (LDFA). **F** Medial proximal tibial angle (MPTA)

**Fig. 2:**
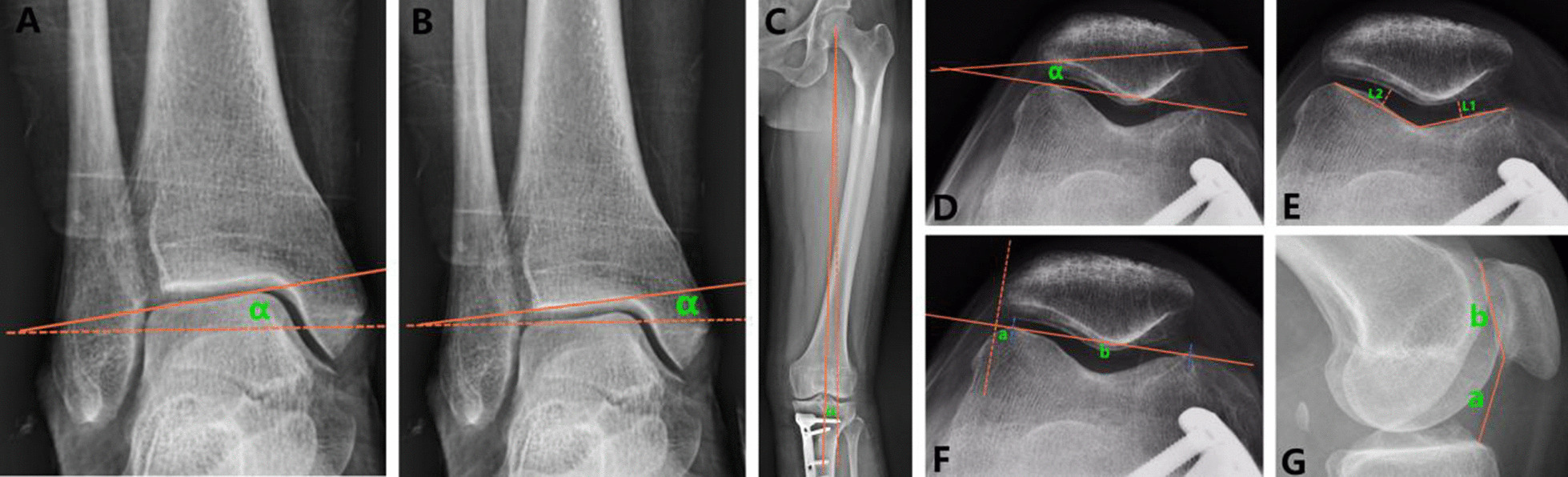
Imaging indexes of ankle, hip, and patellofemoral joints were measured. **A** Talar inclination angle (TIA). **B** Tibial plafond inclination (TPI). **C** Hip abduction angle (HAA). **D** Lateral patellar tilt (LPT). **E** Lateral patellar shift (LPS). **F** Medial patellofemoral space (*L*1) and lateral patellofemoral space (*L*2). **G** Carton–Deschamps index

HKA represents the angle formed between the mechanical axis of the femur and the mechanical axis of the tibia, denoted by (+) for valgus and (−) for varus (Fig. 1A). WBLR is the horizontal distance from the load-bearing line to the inside edge of the tibial plateau (a) divided by the width of the tibial plateau (b) (Fig. 1B). PTS is the angle between the central anatomic axis of the proximal tibia and the tangent line of the tibial plateau (Fig. 1C). JLCA is the angle between the tangent line of the femoral condyle and the tibial plateau, with the positive value indicating lateral joint opening (Fig. 1D). LDFA is the lateral angle formed between the mechanical axis of the femur and the tangential line of the femoral condyle (Fig. 1E). MPTA is the medial angle formed between the mechanical axis of the tibia and the tangent line of the tibial plateau (Fig. 1F). TIA is defined as the angle between the articular surface of the talus and the horizontal line [9] (Fig. 2A). TPI is the angle between the tangent line of the distal tibia and the horizontal line (Fig. 2B). HAA is the angle between the mechanical axis of the femur and the line perpendicular to the ground, annotated by abduction (+) and adduction (−) [1] (Fig. 2C). LPT is the angle formed by the line between the highest point of the internal and external condyle of the femur and the maximum transverse extension line of the patella (Fig. 2D). LPS is defined by drawing a straight line from the highest point of the internal and external condyle of the femur, measuring distance b, and perpendicular to the line at the lateral border of the patella (Fig. 2E). The vertical line is drawn through the highest point of the lateral condyle of the femur, and the distance between the two vertical lines is a; then, LPS = a/b. Medial patellofemoral space and lateral patellofemoral space [10] are the distances between the medial and lateral joint spaces of the patellofemoral joint measured from the center of the joint surface to the patella by drawing vertical lines (Fig. 2F). The Carton–Deschamps index is the ratio of the distance from the inferior pole of the articular surface of the patella to the anterior upper corner of the tibial plateau (a) to the length of the articular surface of the patella (b) (Fig. 2G). The judgment criteria of Kellgren–Lawrence classification were consistent with those in the previous literature [4].

### Statistical analysis

Statistical analyses were performed utilizing SPSS 25.0 software. Normal distribution was assessed for both groups of data, and measurement data conforming to normal distribution were expressed as mean ± standard deviation. The t-test was employed to compare imaging indicators and other measurement data, with the paired t-test utilized for assessing differences between preoperative and final follow-up measurements within each group. Gender and complications data were presented as the number of cases (%), and intergroup comparisons were conducted using the Chi-square test. A significance level of *P* ≤ 0.05 denoted a statistically significant difference.

## Results

### Comparison of general data with radiological parameters

The mean follow-up time for all patients was 79.8 ± 12.15 months, with a mean age of 58.63 ± 4.85 years, encompassing 19 males and 67 females. The average weight-bearing line ratio (WBLR) in the overcorrected group was 70.65 ± 4.58°, compared to 57.48 ± 2.87° in the normal group. No significant differences were observed in age, sex, body mass index, disease duration, operation time, and follow-up time between the two groups (*p* > 0.05), as detailed in Table 1.

**Table 1:** Demographic parameters and clinical characteristics of normal group and overcorrection group

Parameter	Overcorrection group	Normal group	*t/χ*^*2*^	*P*
Age (years)	59.63 ± 3.42	58.34 ± 5.17	1.279	0.207
Gender (Male/female, n)	6/13	13/54	1.275	0.259
BMI (Kg/m^2^)	27.17 ± 2.07	26.83 ± 2.59	0.533	0.595
Course of disease (Years)	5.63 ± 1.8	5.78 ± 2.47	− 0.273	0.813
Time of operation (Hours)	79.05 ± 10.07	80.01 ± 12.73	− 0.303	0.763
Follow-up time (Months)	71.26 ± 3.71	73.06 ± 5.79	− 1.277	0.205
Kellgren–Lawrence stages (III/IV)	14/5	54/13	0.427	0.513

*BI* Body mass index, Mean ± standard deviation. *P < 0.05 is considered significant

At the last follow-up, the medial proximal tibial angle (MPTA) for the overcorrected group was 94.74 ± 0.95°, contrasting with 92.66 ± 2.58° in the normal group, and this difference was statistically significant (*t* = 5.444, *P* < 0.001). The hip–knee–ankle angle (HKA) was 7.26 ± 1.47° in the overcorrected group and 5.15 ± 1.00° in the normal group, showing a statistically significant difference (*t* = 5.874, *P* < 0.001). Hip abductor angle (HAA) in the overcorrected group was − 2.44 ± 1.98°, while in the normal group, it was − 2.44 ± 1.98°, indicating a statistically significant difference (*t* = 2.32, *P* = 0.023). Although the overcorrected group exhibited a higher postoperative correction degree for tibial plafond inclination (TPI) and talus inclination angle (TIA) than the normal correction group, this difference was not statistically significant (*p* > 0.05). Other imaging indicators did not yield statistically significant differences (Tables 2, 3, and 4).

**Table 2:** Measurement of lower limb lineal angle before and at the last follow-up in the normal and overcorrected groups

Parameter	Overcorrection group	Normal group	*t*	*P*
HKA (°)				
Preoperative	− 6.06 ± 1.55	− 5.66 ± 1.71	− 0.912	0.364
Final follow-up	7.26 ± 1.47	5.15 ± 1.00	5.874	< 0.001*
*WBLR (%)*				
Preoperative	25.37 ± 4.59	29.58 ± 9.19	− 2.737	0.008*
Final follow-up	70.65 ± 4.58	57.48 ± 2.87	11.892	< 0.001*
*JLCA* (°)				
Preoperative	4.59 ± 1.6	4.96 ± 1.64	0.875	0.384
Final follow-up	2.66 ± 0.49	2.76 ± 1.49	0.465	0.643
*LDFA *(°)				
Preoperative	88.11 ± 1.35	88.34 ± 1.92	− 0.604	0.549
Final follow-up	87.79 ± 0.62	88.13 ± 1.27	− 1.776	0.081
*MPTA* (°)				
Preoperative	84.92 ± 2.92	85.8 ± 3.08	−1.112	0.269
Final follow-up	94.74 ± 0.95	92.66 ± 2.58	5.444	< 0.001*
*PTS *(°)				
Preoperative	10.08 ± 1.7	9.79 ± 1.68	0.654	0.515
Final follow-up	9.28 ± 1.76	9.16 ± 2.89	0.225	0.823

*HKA* Hip–knee–ankle angle, *WBLR* Weight-bearing line ratio, *MPTA* Medial proximal tibial angle, *LDFA* Lateral distal femoral angle, *JLCA* Joint line convergence angle, and *PTS* Posterior tibial slope; Mean ± standard deviation. *P < 0.05 is considered significant

**Table 3:** Imaging indexes of hip and ankle in the normal and overcorrected groups before and at the last follow-up

Parameter	Overcorrection group	Normal group	*t*	*P*
*HAA *(°)				
Preoperative	2.06 ± 1.72	2.29 ± 1.71	− 0.515	0.608
Final follow-up	− 2.44 ± 1.98	− 1.16 ± 2.1	2.32	0.023*
*TPI *(°)				
Preoperative	5.54 ± 2.6	5.47 ± 5.84	0.077	0.939
Final follow-up	0.54 ± 5.01	2.66 ± 4.3	− 1.823	0.072
*TIA* (°)				
Preoperative	7.24 ± 2.8	6.5 ± 5.33	0.812	0.42
Final follow-up	1.71 ± 5.8	3.02 ± 4.62	− 0.912	0.37

*HAA* Hip abduction angle *TPI* Tibial plafond inclination, and *TIA* Talar inclination angle; Mean ± standard deviation. *P < 0.05 is considered significant

**Table 4:** Imaging measurements of patellofemoral joint in the normal and overcorrected groups at preoperative and final follow-up

Parameter	Overcorrection group	Normal group	*t*	*P*
*LPT *(°)				
Preoperative	10.72 ± 3.16	10.59 ± 2.67	0.173	0.863
Final follow-up	11.05 ± 2.54	10.78 ± 2.29	0.435	0.664
*LPS *(%)				
Preoperative	9.73 ± 3.64	9.47 ± 3.71	0.27	0.788
Final follow-up	10.67 ± 3.64	10.27 ± 3.32	0.458	0.648
*Carton–Deschamps index*				
Preoperative	0.94 ± 0.08	0.93 ± 0.11	0.291	0.771
Final follow-up	0.83 ± 0.05	0.83 ± 0.1	0.373	0.711
*Medial patellofemoral space* (mm)				
Preoperative	5.69 ± 2.86	5.24 ± 2.62	0.652	0.516
Final follow-up	5.98 ± 2.98	5.42 ± 2.72	0.775	0.44
*Lateral patellofemoral space* (mm)				
Preoperative	3.16 ± 1.72	2.93 ± 1.97	0.451	0.653
Final follow-up	3.32 ± 1.52	3.13 ± 1.79	0.418	0.677

*LPT* Lateral patellar tilt and *LPS* Lateral patellar shift; Mean ± standard deviation. *P < 0.05 is considered significant

### Comparison of postoperative complications and clinical function scores

Postoperative complications manifested in five cases in the overcorrection group, including delayed fracture union in one case, joint stiffness in one case, and lateral hinge fracture in 3 cases (26.32%). In the normal correction group, complications occurred in four cases, comprising one case of lower extremity deep vein thrombosis, one case of incision infection, and two cases of lateral hinge fracture, with an incidence of 5.97%. A statistically significant difference in postoperative complications between the two groups was observed (*χ*^2^ = 4.548, *p* = 0.033), as outlined in Table 5. At the last follow-up, no significant differences were found in Hospital for Special Surgery (HSS) and Western Ontario and McMaster Universities Osteoarthritis Index (WOMAC) scores between the two groups (*p* = 0.417 and *p* = 0.691), as indicated in Table 6.

**Table 5:** Comparison of postoperative complications between the normal and the overcorrected groups at the last follow-up

Parameter	Overcorrection group	Normal group	*χ*^*2*^	*P*
Anchylosis	1	0		
Deep venous thrombosis	0	1		
Non-union or delayed union	1	0		
Infection of incisional wound	0	1		
Lateral hinge fracture	3	2		
Total quantity	5/19	4/67	4.548	0.033*

Mean ± standard deviation. * *P* < 0.05 is considered significant

**Table 6:** Comparison of functional scores between the normal and the overcorrected groups at the last follow-up

Parameter	Overcorrection group	Normal group	*t*	*P*
*HSS*				
Preoperative	69.47 ± 6.64	60.52 ± 6.34	− 0.629	0.531
Final follow-up	88.53 ± 4.02	89.31 ± 3.63	0.815	0.417
*WOMAC*				
Preoperative	73.32 ± 3.38	75.88 ± 4.46	0.393	0.695
Final follow-up	24.21 ± 2.07	23.99 ± 2.21	0.398	0.691

*HSS* The American Hospital for Special Surgery score and *WOMAC* The Western Ontario and McMaster University Osteoarthritis Index; Mean ± standard deviation. * *P* < 0.05 is considered significant

## Discussion

Open-wedge high tibial osteotomy (OWHTO) is commonly employed for patients with medial compartment knee osteoarthritis and serves as an effective approach for conservative joint treatment. The realignment of the lower extremity's force line is a pivotal factor influencing clinical efficacy following OWHTO [5]. Precision in force line correction is crucial for the success of OWHTO, and the management of orthopedic angles represents a significant risk factor influencing prognosis [6]. Excessive orthopedic angles may lead to pronounced knee valgus, contributing to lateral compartment wear and degeneration. This phenomenon can compromise the surgical therapeutic effect and potentially elevate the risk of joint replacement therapy. Individualized treatment plans inevitably result in overcorrection, as elucidated in prior investigations involving 34 patients undergoing OWHTO. For those with latent medial laxity or severe varus deformity necessitating substantial correction, inadvertent overcorrection may transpire [11]. The optimal alignment position of the OWHTO force line remains contentious. Hence, this study conducted a follow-up spanning more than 5 years, leading to the following conclusions: Firstly, overcorrection of varus deformity may not significantly impact clinical outcomes within the initial 5 years post-OWHTO but could escalate the incidence of postoperative complications. Secondly, radiographic analysis revealed varying changes in the hip joint, patellofemoral joint, and ankle joints, with the compensatory adduction of the hip joint being the most noteworthy finding.

This investigation observed a significant reduction in TPI and TIA post-OWHTO, with the ankle joint on the affected side tending toward a horizontal or even everted position. This aligns with findings in the previous literature [9, 12], where successful treatment for knee and ankle osteoarthritis after high tibial osteotomy was reported [13]. As an integral part of the lower limb force line, alterations in the knee joint force line inevitably induce compensatory changes in adjacent joints. Although the postoperative correction degree of TPI and TI in the overcorrection group was higher than that in the normal correction group, the difference was not statistically significant (*p* > 0.05). A parallel study found no significant difference in postoperative TIA between the over-MPTA correction group and the normal correction group (*p* = 0.777) [1]. While our results align with this study, further research is warranted to ascertain whether the degree of ankle valgus worsens with an extended follow-up. Additionally, overcorrecting varus deformity was noted to lead to an increased compensatory adduction of the affected hip joint. The previous research has demonstrated a significant preoperative increase in the adduction moment of the hip and knee joints in patients with valgus alignment following high tibial osteotomy. Postoperatively, this adduction moment decreases to levels below the knee, while the hip joint returns to a normal level. When the knee exhibits slight valgus, frontal plane torque around the hip can be reduced to a normal level, potentially benefiting patients with ipsilateral hip and knee osteoarthritis [14]. Furthermore, compensatory hip joint motion ensures that the knee joint line tilt angle remains parallel to the ground post-OWHTO. A certain degree of overcorrection of MPTA does not seem to influence clinical outcomes after OWHTO [1]. Given the excessive displacement of the weight-bearing axis of the affected limb, the intensified compensatory adduction of the hip is considered rational for maintaining lower limb stability and facilitating the force line's re-remodeling.

While the overcorrection of varus deformity did not yield significant alterations in patellar joint position, our study contributes valuable data indicating noteworthy patellar downward displacement with lateral shifting subsequent to OWHTO. Simultaneously, our results affirm the finding that internal and lateral patellar spaces diminish after OWHTO, aligning with the observations of Ishimatsu et al. [10]. The previous investigations have presented conflicting perspectives on whether OWHTO induces patellar descent, tilt, and lateral displacement. Some studies suggest that OWHTO can lead to patellar sagging, reducing the Carton–Deschamps index by 1.7% [15]. Another study involving 18 knees reported an average 15% reduction in patellar bone height post-OWHTO [16]. The suggested mechanism involves the wedge opening on the tibial tubercle, which pulls it distally, subsequently causing patellar shift toward the distal end. Additionally, the proximity of the osteotomy to the patellar tendon may induce cicatricial contracture post-surgery, contributing to a decrease in patellar height. However, a retrospective study of 62 knee joints found no significant alteration in patellar tilt and displacement following OWHTO through radiological evaluation [17]. In contrast, recent studies indicate a decrease in LPT from 8.67 ± 2.60 degrees pre-surgery to 6.13 ± 2.30 post-surgery, signifying lateral patellar displacement and internal rotation of the distal tibia as crucial factors contributing to LPT reduction [18]. Our present study may offer novel data and theoretical support to elucidate changes in patellar position after OWHTO.

A growing body of the literature reports on the incidence of complications and revisions post-OWHTO [19–22]. The previous studies have found that the 5-year survival rate of prostheses after OWHTO is 80% [19], and the overall complication rate is 63.7% [20]. The most common adverse event was delayed healing (12%) [21]. Patients with a loss of correction angle are associated with a greater body mass index (BMI) [22]. A recent meta-analysis involving 7836 patients [23] revealed a total complication rate of 6.9% after high tibial osteotomy (HTO). The most common intraoperative complication was lateral hinge fracture (9.1%), while superficial infection was the predominant postoperative complication (2.2%). The correction failure rate stood at 1.2% and the implantation failure rate at 1.0%. The overcorrection group exhibited a heightened incidence of complications, notably an increased risk of lateral hinge fractures. In this group, the osteotomy space notably expanded, and greater bone defects corresponded to a higher incidence of postoperative osteotomy non-union or delayed union. Prior studies also corroborate [24] that an opening width exceeding 13.0 mm can lead to delayed bone healing after OWHTO. Furthermore, an enlarged osteotomy space, increased intraoperative fluoroscopy times, and bone grafting in some patients extended the operation time. The prolonged operation time, coupled with augmented intraoperative bleeding and prolonged exposure of the operative area to air, elevated the risk of infection. These factors collectively contribute to the heightened incidence of postoperative complications in the overcorrection group.

In terms of clinical efficacy, Myrnerts observed that patients with excessive correction exhibited significantly better outcomes than those with normal correction [8]. Dugdale et al. [7] proposed that the highest postoperative clinical efficacy satisfaction was achieved when the lower limb's negative gravity line passed through 62.5% of the tibial plateau width following medial high tibial osteotomy. Another international study evaluated the hip–knee–ankle angle, achieving an average correction of the affected limb from − 4.3 to 3.7°, allowing patients to regain excellent function and engage in daily physical activities [25, 26]. Currently, it is generally accepted that setting different intraoperative force lines based on various knee wear conditions can minimize medial ventricular pressure in the knee joint, thereby alleviating knee pain [27, 28]. As the knee HSS score is influenced by factors such as joint motion and muscle strength, the lower limb's force line and cartilage repair exert more influence on pain improvement and daily motor function; hence, the score improvement is not statistically significant [29, 30]. To date, HSS scores and WOMAC scores have not shown significant changes in the overcorrected group, with no notable decline in knee function observed in patients overcorrected for at least 5 years of follow-up. However, a substantial orthopedic angle and overcorrection of the lower limb force line are likely to induce excessive knee valgus, leading to wear and degeneration of lateral compartment cartilage and increasing the likelihood of postoperative lateral compartment degeneration in the knee joint. Consequently, the long-term clinical effects on the knee joint and whether they elevate the risk of knee joint replacement require extended follow-up.

### Strengths and limitations

We conducted a 5-year follow-up study with a substantial number of radiographic measures. However, there were several limitations in this study, including: (1) No knee joint X-ray can guarantee absolute neutrality, and minor rotation, tilt, and internal and external inversion may introduce substantial errors in subsequent measurements. (2) As a single-center retrospective study, this study still has the limitation of a small sample size, and multi-center prospective studies are still needed in the future. (3) We only conducted an analysis of limited clinical data, and more imaging indexes need to be collected and analyzed in our future studies.

## Conclusion

Overcorrection of varus deformity may not significantly impact clinical outcomes within 5 years post-OWHTO but may elevate the incidence of postoperative complications and lead to increased compensatory adduction of the hip.

## Data Availability

The datasets generated during and/or analyzed during the current study are available from the corresponding author on reasonable request.

## Abbreviations

OA: Osteoarthritis
OWHTO: Open-wedge high tibial osteotomy
WBLR: Weight-bearing line ratio
HSS: American Hospital for Special Surgery
WOMAC: Western Ontario and McMaster Universities Osteoarthritis Index
HKA: Hip–knee–ankle angle
MPTA: Medial proximal tibial angle
LDFA: Lateral distal femoral angle
JLCA: Joint line convergence angle
PTS: Posterior tibial slope
HAA: Hip abductor angle
TPI: Tibial plafond inclination
TIA: Talus inclination angle
LPT: Lateral patellar tilt
LPS: Lateral patellar shift
